# How social network heterogeneity facilitates lexical access and lexical prediction

**DOI:** 10.3758/s13421-016-0675-y

**Published:** 2016-11-28

**Authors:** Shiri Lev-Ari, Zeshu Shao

**Affiliations:** Max Planck Institute for Psycholinguistics, Postbus 310, 6500 AH Nijmegen, The Netherlands

**Keywords:** Social networks, Variability, Lexical access, Prediction, Language production

## Abstract

People learn language from their social environment. As individuals differ in their social networks, they might be exposed to input with different lexical distributions, and these might influence their linguistic representations and lexical choices. In this article we test the relation between linguistic performance and 3 social network properties that should influence input variability, namely, network size, network heterogeneity, and network density. In particular, we examine how these social network properties influence lexical prediction, lexical access, and lexical use. To do so, in Study 1, participants predicted how people of different ages would name pictures, and in Study 2 participants named the pictures themselves. In both studies, we examined how participants’ social network properties related to their performance. In Study 3, we ran simulations on norms we collected to see how age variability in one’s network influences the distribution of different names in the input. In all studies, network age heterogeneity influenced performance leading to better prediction, faster response times for difficult-to-name items, and less entropy in input distribution. These results suggest that individual differences in social network properties can influence linguistic behavior. Specifically, they show that having a more heterogeneous network is associated with better performance. These results also show that the same factors influence lexical prediction and lexical production, suggesting the two might be related.

Languages allow the same events to be described in myriad ways. Some manners of description are more common than others. For example, the same piece of furniture can be referred to as either a *dresser* or a *chest of drawers*, but the former is more common in American English (Yoon et al., 2004). Occasionally, a term’s frequency depends on the characteristics of the speaker. That is, in some cases, lexical choice among parallel terms does not reflect shades of meaning of the referent, but it does, on the other hand, reflect a characteristic of the speaker. For example, *bicycle* and *bike* can refer to the same object, but the former is more likely to be used by older adults than by younger ones (Yoon et al., 2004).

Knowing how lexical choice is distributed across the population can be useful in language processing. After all, an integral aspect of language processing is prediction of upcoming information, and predictions are often specific enough to consist of specific words (e.g., Balota, Pollatsek, & Rayner, 1985; Delong, Urbach, & Kutas, 2005; Pickering & Garrod, 2013). Previous research shows that reading an article that does not agree with the anticipated following noun (e.g., reading the article, *an*, when expecting the noun *kite*) leads to an N400 effect whose amplitude is larger the more anticipated the mismatching noun is (e.g., Delong et al., 2005). This finding shows that people predict specific words. Correspondingly, predictable words are integrated more easily, as evidenced, by example, by shorter reading times (e.g., Balota et al., 1985). The goal of this article is to test whether properties of our social network influence our knowledge of word distributions and, consequently, our lexical choices and ability to predict others’ lexical choice. Second, it examines whether own lexical choice and prediction of others’ lexical choice are influenced by the same properties of our social network; they thus shed some light on the relation between the representations used for prediction of others’ speech and the representations used for own speech production.

## The influence of input variability on learning

How might the properties of our social network influence our language use and our ability to predict others’ use? The key factors are likely to be input variability and input representativeness. People differ in the size of their network. Some people might interact with only a few other people while others might have a much wider network. For example, Hill and Dunbar (2003) found that the number of Christmas cards people send out can vary from fewer than 25 to more than 350. Importantly, exposure to more people has been shown to boost learning. For example, Japanese speakers succeed better at acquiring the phonological contrast between/r/and/l/if they are trained with speech from five speakers than if they are trained with speech from a single speaker, even when the amount of speech is slightly higher in the single-speaker condition (Lively, Logan, & Pisoni, 1993; also see Sadakata & McQueen, 2013, for similar results). Similarly, 14-month-old infants often fail at recognizing that/buk/and/puk/are different words (and thus that/b/and/p/are contrastive phonemes). However, such infants succeed at recognizing this difference if they are exposed to 18 speakers rather than a single speaker repeating one of these words, even when the amount of exposure is identical (Rost & McMurray, 2009). Adaptation to foreign-accented speech is similarly better with exposure to several speakers compared to one, even when the amount of exposure is held constant across conditions (Bradlow & Bent, 2008). The boost to learning that exposure to multiple speakers provides is argued to be due to the greater variability in input from multiple speakers compared to a single speaker. Correspondingly, Sumner (2011) showed that exposure to multiple tokens from the same speaker leads to better learning than exposure to the same token of the speaker the same number of times.

Positive effects of variability, and number of speakers in particular, are not restricted to the phonological level. Thus, native speakers of American English learn Spanish words better if they are exposed to six speakers producing the words rather than a single speaker repeating the words six times (Sommers & Barcroft, 2006), and individuals who have had multiple interlocutors with whom they spoke a heritage language are more proficient in it than those who have had fewer interlocutors, even after controlling for differences in frequency of interaction (Gollan, Starr, & Ferreira, 2015). Variability along other dimensions has also been shown to improve learning. For example, both infants and adults are better at learning grammatical patterns when exposed to many exemplars rather than a few exemplars repeated the same number of times (Eidsvåg, Austad, Plante, & Asbjørnsen, 2015; Gómez, 2002), and some have even argued that the positive effect of frequency on learning is in fact due to the correlation between frequency and context variability (Adelman, Brown, & Quesada, 2006). But why does variable input help? Several accounts have been proposed, including the suggestion that during the formation of new categories, variable input might assist in learning which aspects of the input are relevant for categorization and which ones are not. Indeed, Rost and McMurray (2010) showed that varying the irrelevant prosodic and indexical aspects of the input, such as pitch and intonation, boosts learning of a phonological contrast based on voice onset time difference while varying the input along the relevant aspect, voice onset time, did not facilitate learning. In the case of learning the distribution of lexical terms and their conditional dependency on speaker characteristics, variability might boost learning by highlighting which indexical properties are predictive of lexical choice and which ones are not. For example, receiving input from both women and men of various ages could allow a listener to learn that gender is not predictive of lexical choice for a certain concept, and that lexical representations can therefore be generalized across genders, but that age is predictive of lexical choice for that concept. In general, to the degree that the variability in the input reflects the variability in the population, more variable input would be more representative and more informative about patterns in the population and reflect them more reliably.

A slightly different account would suggest that the representativeness of variable input facilitates performance not because it teaches individuals about patterns in the population they can predict but because it changes their own patterns of use and representations, and these representations are used to predict others’ use. This account fits with two different strands of evidence in the literature. First, some accounts of language processing propose that processing involves constructing forward models of simulated production. That is, listeners are assumed to covertly simulate the continuation of others’ speech, thus facilitating its processing and leading to alignment between interlocutors (Pickering & Garrod, 2013). Second, research in social psychology indicates that when people predict others’ behavior and preferences, they start out by anchoring in their own behavior and preferences, and then adjusting a little, often, insufficiently so (Ross, Greene, & House, 1977). The studies in this article provide an initial response to the question of how variability might help prediction and its relation to its influence on production. The first study tests whether the properties of individuals’ social network are related to their ability to (explicitly) predict others’ lexical choice. The second study examines whether the properties of individuals’ networks influence lexical access and their own lexical choice in the same manner. The third study uses simulations to further examine the relation between network properties and lexical access.

Additionally, the studies in this article investigate which aspects of the social network predict performance. In general, different properties of the network could influence its variability. The previously mentioned studies at the phonological level manipulated variability by varying the number of speakers during exposure (Bradlow & Bent, 2008; Lively et al., 1993; Rost & McMurray, 2009). Therefore, the size of people’s social network might influence the variability of their input. At the same time, social networks might be larger without being more variable along dimensions that are relevant for lexical choice. One factor that influences lexical choice is age. Therefore, in this study we also examined whether the age variability in people’s social network is related to network size, and whether it influences performance. Last, another aspect that could influence a network’s variability is its density. Denser networks—that is, networks in which many members are connected to many other members in the network—are likely to be less variable. The reason for that is that people tend to align their language use to that of their interlocutors (e.g., Giles, Coupland, & Coupland, 1991). Such alignment has been argued to lead to long-term changes in representation and use (e.g., Pardo, Gibbons, Suppes, & Krauss, 2012). Therefore, network members that are connected to other network members provide less new and independent information than less connected members, and are therefore less likely to increase variability compared to unconnected members. It is also possible that properties of the network are not independent of one another, and that, for example, larger social networks tend to be less dense and more variable in terms of age. Thus, the different properties might correlate and reinforce each other. The studies in this article examine the relative role of each of these properties of the social network. Specifically, Study 1 examines network size and network age variability; Study 2 examines all three properties, namely, network size, network age variability, and network density; and Study 3 focuses on the effect of age heterogeneity that was found in Studies 1 and 2.

## Study 1

Study 1 tests whether the properties of individuals’ social network influence their ability to predict speakers’ lexical choice, and in particular, their sensitivity to the variation of lexical choice across ages. To do so, participants were asked to estimate the term that 19- to 25-year-old college students are most likely to use to refer to a depicted object, and the term that 60- to 75-year-olds are most likely to use to refer to the same object. The accuracy of participants’ estimates was assessed by comparing their responses to norms for the same pictures for these two age groups. We then examined whether properties of participants’ social network predict the accuracy of participants’ estimates.

Our measure of lexical prediction is novel. Unlike more conventional measures of lexical prediction, such as fixation on objects that are likely to be referred to next, our measure focuses on the aspect of lexical prediction that is likely to be influenced by social network size—knowing the dependencies of lexical use on speaker characteristics—while minimizing the influence of other factors that play a role during lexical prediction but are unlikely to be influenced by social network size, such as ability to integrate the context during online language processing (e.g., Federmeier & Kutas, 2005).

### Method

#### Participants

Ninety-five participants with USA IP addresses were recruited via Mechanical Turk (www.mturk.com). Three participants performed the task more than once, and therefore, their second session was excluded. One participant was excluded because he or she reported being a nonnative speaker of English, and one participant was excluded for reporting being under 18 years of age. Analyses were therefore over the remaining 90 participants.

#### Stimuli

Thirty-three pictures were selected from the set used in the picture naming norm study by Yoon et al. (2004). In that study, picture naming norms were gathered for two groups of participants: college students (ages 19 to 25 years) and older adults (ages 60 to 75 years). We selected from these norms pictures which had relatively low naming agreement. Specifically, the pictures we selected all had naming agreement lower than 75% for at least one of the age groups (range: 32.7%–94.7%; *M* = 63.7%, *SD* = 16.9), meaning that at most 75% of the respondents provided the most common response. Additionally, we only selected pictures for which most of the variance was due to use of synonyms rather than misidentification of the depicted object in the picture. For each picture, we asked participants to write down the most common way that (i) a college student would refer to the picture, and (ii) the most common way that an older adult (60–75 years old) would refer to the picture. Because of an error, the first 15 participants were asked for two separate age-specific responses for only 20 of the pictures, so only these responses were included in the analysis. The other 75 participants provided age-specific responses to all 33 pictures. Note that not all pictures are named differently by people of different ages. Thus, errors could emerge from lack of knowledge of the appropriate label for any age group, from underestimation of differences among age groups or from overestimation of differences across age groups.

All participants were also asked about their linguistic exposure. The first 55 participants responded to a lengthy questionnaire that included many exploratory questions that were gathered for future research on network structure. The other 40 participants were only asked a subset of the questions that were of interest for this study, namely, how many people they talk to in a regular week, the age range of the people with whom they converse most often, and the number of hours they talk per week. Participants’ responses regarding the age range of their frequent interlocutors were then entered as the age difference. For example, if a participant reported that the age range of their frequent interlocutors was 25–30, 5 was entered as the age range. Participants were also given the choice of indicating that there was no particular age group with which they interact. In that case, the difference between the ages of their oldest and youngest interlocutor in a typical week was calculated.

#### Procedure

Participants first answered the questionnaire about their linguistic interactions, and then performed the reference prediction task. Pictures were presented in one fixed-random order, with the two age-specific questions about each picture appearing consecutively, starting with the question about the college-aged speakers. Participants performed the task at their own pace.

### Results

Participants’ responses were scored as correct if they provided the most common label according to the norms by Yoon et al. (2004) and as an error otherwise. Participants’ social network size ranged from 1 to 100 (*M* = 17, *SD* = 20). The common age range of the people with whom they interact ranged from 1 to 58 (*M* = 16, *SD* = 15). Four participants were excluded from analysis for having a network size more than 2.5 standard deviations away from the mean (>70). Without these participants, network size ranged from 1 to 60 (*M* = 13, *SD* = 12), and interlocutors’ age ranged from 1 to 58 (*M* = 15, *SD* = 15). To test whether network size and network age variability influence lexical prediction accuracy, we submitted the accuracy (coded as 0 or 1) to a logistic regression analysis using mixed effects models with crossed random effects for participants and items (see Baayen, Davidson, & Bates, 2008, for detail). The model included Participants and Items as random factors, and Picture Agreement, Network Size, Age Range, Question’s Age Group (young, old), and the interaction between Network Size and Question’s Age Group and Age Range and Question’s Age Group as fixed factors. The model included intercepts and all possible slopes for all random factors. All continuous variables were centered, and Question’s Age Group was dummy coded with “old” as the baseline. Picture Agreement is the proportion of participants in Yoon et al.’s (2004) study who provided the most common response. It was entered to account for the greater ease of predicting the most common label for pictures with high naming agreement than for pictures with low naming agreement.

**Fig. 1:**
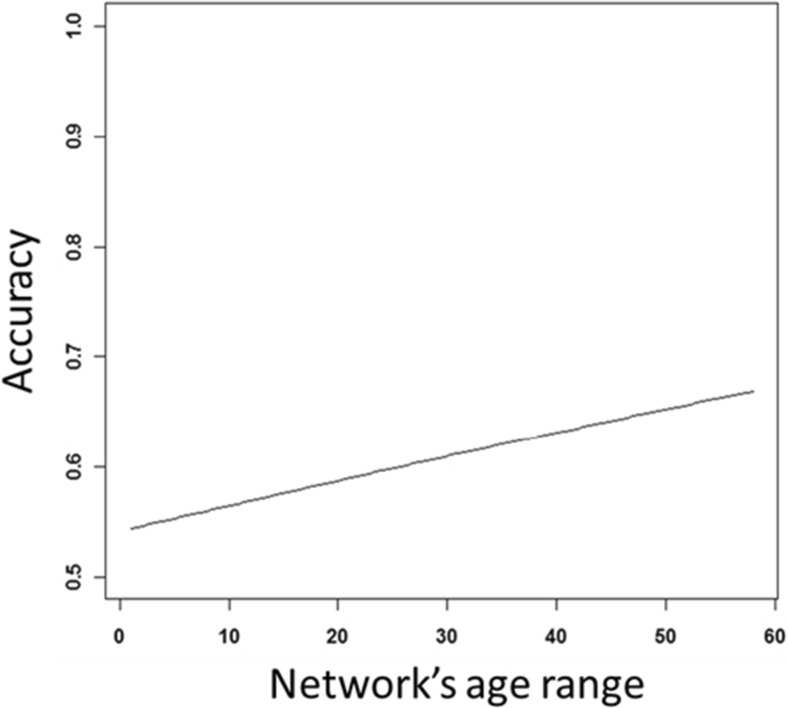
The effect of network’s age range on lexical prediction accuracy

The model revealed an effect of Picture Agreement (β = 0.02, *SE* = 0.007, *z* = 2.91, *p* < .01), such that accuracy was higher for pictures with higher name agreement. Question’s Age Group also had an effect (β = 0.96, *SE* = 0.29, *z* = 3.28, *p* < .01), indicating that accuracy was higher for question about college students. This is probably because the majority of our participants were closer in age to college students than to older adults. Seventy five of the participants reported their age. Their average age was 35 years (*SD* = 10.6). Importantly, Age Range also had an effect (β = 0.01, *SE* = 0.003, *z* = 2.76, *p* < .01; see Fig. 1, and Table 1, in the Appendix^1^), indicating that the larger the age range of participants’ frequent interlocutors, the more accurate participants were overall. Specifically, every increase of 5 years in the age range in participants’ social circle led to an improvement of roughly 1% in participants’ prediction. In contrast, participants’ Network Size did not predict their performance.

One question that arises is how participants’ performance depends on the age group of their network, and more importantly, how the role of network properties depends on the similarity between the age range in participants’ network and the age group about which they are asked. In other words, does age variability in one’s network boost lexical prediction equally when predicting lexical choices of speakers who are of similar and of different age than those in one’s network? To examine this, we coded for each participant for each question whether the age group about which the question asks is closer to the age of their common interlocutors than the other age group or farther from it. We then ran a similar logistic mixed-model analysis to the one beforehand, only replacing Question’s Age Group with Question’s Age Similarity to Network Age. The analysis revealed, as before, effects of Picture Agreement (β = 0.03, *SE* = 0.008, *z* = 3.45, *p* < .001), and Age Range (β = 0.008, *SE* = 0.004, *z* = 1.97, *p* < .05), showing that, as before, accuracy was higher when picture name agreement was higher as well as the more heterogeneous the ages in participants’ network were. Importantly, Age Range did not interact with Question’s Age Similarity to Network Age, indicating that having greater age heterogeneity in one’s network is useful whether one is predicting the lexical choice of people of a similar age to the ones in one’s network or whether one is predicting lexical choice for people of a different age than the age of one’s network members. Surprisingly, there was also an interaction between Network Size and Question’s Age Similarity to Network Age (β =- 0.02, *SE* = 0.01, *z* = -2.06, *p* < .04; see Table 2 in the Appendix for the full results), yet a further examination of the simple effects driving the interaction showed that neither was significant (both *p*s > .1).

The results of this study then show that the properties of one’s social network influence one’s lexical skills. Specifically, they show that having a more variable social network is helpful. Participants who interact with people of different ages are better overall at predicting how people of different ages refer to objects, regardless of whether their social network includes people of these ages. As mentioned earlier, however, production and prediction are related. Therefore, the question remains whether social network properties directly influence prediction or whether social network properties influence production, and only consequently, prediction. Study 2 therefore examines the influence of the properties of people’s social networks on their own lexical choice and representations.

## Study 2

Study 2 tests whether the properties of people’s social network influence their linguistic representations and lexical choice in a manner similar to the one that they influence their lexical prediction. To do that, participants in Study 2 named a set of pictures similar to the one used in Study 1. If properties of the social network improve lexical prediction by providing a more representative sample of the population, then they might influence lexical access, as reflected in response times. If social network properties improve lexical prediction by influencing one’s own lexical choices, then we might also find that network properties influence how normative participants’ lexical choices are.

Study 2, unlike Study 1, was conducted in The Netherlands. Therefore, in order to code the normativity of participants’ responses, we needed to first assess what the normative responses for the items are. To do so, we gathered norms for these items with a representative sample of the Dutch adult population. These norms were not collected from our ordinary sample pool, but from the wider Dutch population using a private company that specifically specializes in recruiting respondents according to desired demographics—in our case, a sample that is representative of the adult Dutch population in terms of age and gender. Thus, we ensured that our norms reflect the lexical distribution in the adult Dutch population at large.

Additionally, Study 2 used a more focused and detailed social network questionnaire. Informed by the results of Study 1, the questionnaire in this study only asked about oral interactions, but gathered more details about them. Specifically, participants listed all their weekly interlocutors and provided information about them. This allowed us to gather, in addition to the total number of interlocutors, a more precise estimate of the age variability in the network, as well as information about the density of the network, providing a comprehensive view of the role of social network properties in lexical choice.

### Method

#### Participants

Ninety-nine native Dutch speakers participated in the study. Three participants were excluded because the density of their social network was more than 2 standard deviations away from the mean, so analyses were carried over the remaining 96 participants.

#### Tasks

Social network questionnaire. Participants were asked to list all the people that they talk to for at least 5 minutes every week. Participants were instructed to only include interlocutors who are native Dutch speakers above the age of 12. Additionally, participants were told that if they regularly talk to specific types of people whose identity changes from week to week (e.g., clients), they should list them as well. For each person participants listed, they were asked to provide several details, including age. Once participants completed the list, the software generated a list of all possible pairs of network members. Participants needed to indicate all the pairs of interlocutors who regularly interacted with one another. Participants network size was calculated as the total number of people they listed (range: 5–60, *M* = 22). Age variability was calculated as the standard deviation of the ages in participants’ network (age *SD* range: 2–22, *M* = 13). Network density was calculated as the proportion of interacting pairs of members out of all possible pairs (range: .07–.4, *M* = .23). See Table 3 in the Appendix for their correlations.

Naming task. We selected 107 target pictures from Druks and Masterson (2000), Bates et al. (2003), and the picture databases (http://www.pixelio.de/and http://pixabay.com/en/). Most of these pictures (*N* = 78) were also included in Dutch norms conducted in Belgium (Severens, Van Lommel, Ratinckx, & Hartsuiker, 2005). We selected pictures that, according to the norms and previous studies at our lab, were likely to elicit more than one appropriate name in Dutch. Additionally, we included as control 10 pictures from those sources that had 100% name agreement according to Severens et al.’s (2005) norms.

Pictures were presented one at a time in one of four fixed random orders. Each picture remained on the screen until participants responded or until 3 seconds had elapsed. The intertrial interval was 1,000 ms. The task was carried out in a sound attenuated booth, and responses were recorded using a voice key.

#### Procedure

Participants first filled out the social network questionnaire. Afterwards, they performed the naming task.

#### Norm collection

The norms were collected after all participants were run. As explained below in the Data Coding section, six of the pictures turned out to have 100% name agreement. Additionally, one picture had 99% name agreement. Therefore, norms were collected for the remaining 100 pictures. We divided this set of 100 pictures into two sets of 50 pictures. We then hired the services of a company focusing on targeting specific demographics for market research to recruit two samples of participants, one for each subset of the data. Both samples were targeted such that they would be representative of the adult Dutch population in terms of age and sex. The samples were of 126 and 125 participants, respectively. Each participant saw the pictures one at a time in a random order. For each picture, participants were asked to label the picture with a single word.

#### Data coding

First, all responses were coded as appropriate or inappropriate. For example, naming the train tracks *xylophone* was considered erroneous. This led to the exclusion of 511 erroneous responses and no responses (4.3%). A review of the response revealed that six of the target pictures had 100% name agreement despite showing variation in the norms. As they included no variation to analyze, they were excluded from all analyses. Name agreement among the remaining pictures in our sample ranged from 36% to 99% (*M* = 68%). We classified name normativity according to the responses provided by the norms that we collected. The most frequent response provided by participants was always coded as the normative response. All other appropriate names were coded as nonnormative. For nine items, there were two names that were similar in frequency (i.e., <6% difference). Those items were excluded from the lexical choice analysis, as there is no single normative response. These items, however, were included in the response time analysis.

### Results

To test whether the properties of people’s social network influence the normativity of their lexical choice, we ran a logistic mixed-model analysis with Participants and Items as random factors, and Network Size, Age Variability, and Network Density as fixed factors. The model included all possible random slopes. None of the factors significantly predicted normativity (Network Size: |*z*| < 1; Age Variability: |*z*| < 1; Network Saturation: *z* = 1.65, *p* > .05). This suggests that the reason that network properties influence prediction ability is not by influencing one’s own lexical choice. At the same time, the variability in how normative participants was low (*M* = 0.78, *SD* = 0.06), potentially too low to examine individual differences.

Next we tested whether network properties influence linguistic representations, and thus ease of lexical access, as reflected in response times. We therefore ran a mixed-effect model with Participants and Items as random variables, and Network Size, Age Variability, Network Density, Response, and H Index (name entropy) as fixed factors. Response refers to whether the response that participant provided was the dominant or the subordinate response. It was included to control for the fact that dominant responses are retrieved more quickly. The H Index measures the entropy of the name distribution for the specific item, according to the collected norms. It is well established that higher entropy leads to slower lexical access (e.g., Shao, Roelofs, Acheson, & Meyer, 2014; Snodgrass & Vanderwart, 1980). The random structure of the model included intercepts for both random variables, as well as all possible slopes. The dependent measure was participants’ log-transformed response times.

**Fig. 2:**
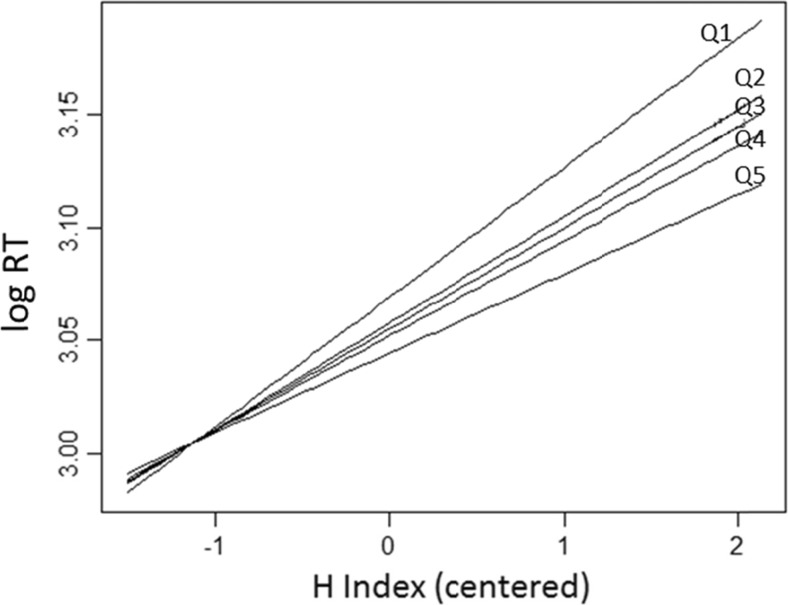
The effect of H Index on response time, as dependent on Age Variability in participants’ network. The *lines* represent the average results per quantile in participants’ distribution of Age Variability

Results showed that, in line with previous literature, participants were faster to name the picture when they used the dominant rather than the subordinate name (β = -75.59, *SE* = 23.64, *t* = -3.20). Participants were also slower to respond the higher the entropy in the name distribution of that item is (β = 223.7, *SE* = 40.51, *t* = 5.52). Importantly, this effect was modulated by network Age Variability, as indicated by an interaction between H Index and Age Variability (β = -4.53, *SE* = 2.16, *t* = -2.10; see Table 4 in the Appendix for the full results^2^). The interaction reflects the fact that higher entropy always slowed that responses, but its effect was less detrimental the more age-varied participants’ network was (see Fig. 2). In other words, having an age-variable network facilitates lexical access mostly when labels are hard to retrieve.

The results of Study 2, then, suggest that the same network variable that influences lexical prediction influences linguistic representation, as reflected in ease of lexical access. Specifically, greater Age Variability in one’s network is associated with both better lexical prediction of others’ lexical choice, and with greater ease of retrieving the names of difficult-to-label pictures. In contrast, network size and network density do not seem to influence lexical choice. So why do people with a more age-heterogeneous network find it easier to access the names of difficult-to-label pictures? And is this related to prediction ability? As a first step to answer this question, we ran network simulations on our collected norms to see how having networks of different age variability influences the name distribution in people’s input.

## Study 3

To investigate how the age variability in people’s networks influences the input they receive, we simulated different networks with the norms we collected and examined the relation between the age variability in the network and the entropy in the name distribution for each item, as reflected in the H Index. We hypothesized that participants with more age-variable networks in Study 2 were faster to access the labels for difficult-to-name items because the entropy in the name distribution in their input is lower. As our norms were collected from two samples, each representative of the Dutch population, we selected one of the two samples at random. The sample then included responses from 126 adult native Dutch speakers to 50 of the items that were used in Study 2.^3^

### Method

We ran 300 simulations, 100 in each of the following three network conditions. In the low age variability condition, we sampled all the responses from 15 randomly selected speakers who are all between the ages of 18 and 34. In the medium age variability condition, we sampled all the responses from 10 randomly selected speakers between the ages of 18 and 34 years, and five randomly selected speakers between the ages of 35 and 54 years. In the high age variability condition we sampled all the responses from five randomly selected people between the ages of 18 and 34 years, five randomly selected speakers between the ages of 35 and 54 years, and five randomly selected speakers that are 55 years or older. For each simulation, we calculated the H Index. Additionally, for each item, we calculated the average name agreement, that is, the proportion of the responses that the most dominant name received across all networks. Name agreement is known to influence difficulty of lexical access, such that items with lower name agreement are more difficult to access (e.g., Shao et al. 2014).

### Results

To test whether network age variability influences name distribution, we ran a mixed-effects model with Simulation and Item as random variable, and Age Variability, Name Agreement and their interaction as fixed factors. Age Variability was coded as an ordinal factor, such that simulations in the Low Age Variability condition were coded as 1, simulations in the Medium Age Variability condition were coded as 2, and simulations in the High Age Variability condition were coded as 3. The random structure of the model included intercepts for the random variables, as well as slopes for Age Variability and for Name Agreement for the Simulation variable, and a slope for Age Variability for the Items variable. The dependent measure was the H Index for each item in each simulation.

Results revealed that a network’s age variability influences the H Index of the responses, such that having greater age variability in one’s network leads to lower entropy (β = -0.16, *SE* = 0.05, *t* = -2.92). This effect, however, is modulated by an item’s Name Agreement, such that Age Variability in the network has a larger effect on the entropy of responses for difficult-to-label items (β = 0.23, *SE* = 0.08, *t* = 2.68). These results explain why in Study 2, having greater Age Variability in a speaker’s network led them to access difficult-to-label names more easily. It shows that having greater Age Variability in one’s network *reduces* the entropy in the name distribution that a speaker is exposed to, and consequently, makes lexical access easier, especially for difficult-to-access words (see Table 5 in the Appendix).

But why does having a more heterogeneous network reduce the entropy? We hypothesize that because language use varies with age, the name distribution varies across ages, including the identity and frequency of the competitors. For example, *geestelijk* (cleric) is a strong competitor for *pastoor* (priest) among middle-aged speakers, but not at other age groups. Therefore, if one’s input is derived out of speakers of different age groups, age-specific competitors pose less competition, rendering the dominant name more dominant. To test if that is the case, we conducted an analysis over the log-transformed name agreement of the most dominant competitor in the simulations we ran. The mixed-model analysis included Item and Simulation as random variables, and Age Variability, H and their interaction as fixed factors. Age Variability was coded as an ordinal factor, as in the previous analysis.

Results showed that, as predicted, having higher age variability in the network leads to less competition, as indicated by lower name agreement for the strongest competitor (β = -0.03, *SE* = 0.01, *t* = -2.94). This effect, however, was modulated by H (β = 0.0.02, *SE* = 0.006, *t* = 3.73), which, on its own, was positively related to competitor’s name agreement (β = 0.14, *SE* = 0.004, *t* = 34.18). The interaction reflects the fact that network age variability reduced competition more for items with low entropy (see Table 6 in the Appendix). It is unclear why the influence of age variability is larger for items of low rather than high entropy, especially considering the fact that age variability reduces H and facilitates response times more for difficult to name items. It is clear though that network age variability boosts lexical access by reducing entropy, and the reduction in entropy seems to be partially due to the fact that age variability reduces the competition that the leading competitors pose.

## General discussion

Language use varies across the population. In the case of lexical choice, many objects and events can be referred to in more than one way. Interestingly, such variation in lexical choice sometimes does not reflect a difference in the meaning of the referent, but, rather, a demographic characteristic of the speaker. Sensitivity to such variation is important, as it can assist language processing. For example, sensitivity to the distribution of lexical terms across different types of speakers can improve one’s ability to predict upcoming words, which, in turn, can facilitate processing. In this article, we examined how properties of people’s social network influence their ability to predict upcoming words, as well as whether it influences their representation and own use. In particular, we focused on aspects of the social network that might influence the variability of the input that people receive, as greater variability might lead to having a more representative sample of the population, allowing more accurate knowledge of linguistic patterns across different subgroups in the population. The three aspects of the network we examined were therefore Network Size, Network Age Variability, and Network Density.

We initially hypothesized that Network Size could lead to increased variability in the network, and thus influence linguistic representations and improve lexical prediction. Indeed, previous studies at the phonological level manipulated input variability by manipulating the number of speakers (Bradlow & Bent, 2008; Lively et al. 1993; Rost & McMurray, 2009). Relatedly, we hypothesized that greater age variability would improve lexical prediction and lead to more normative lexical choice, as Age Variability is a more direct measure of network variability than network size, and focuses on a specific dimension (age) that was specifically relevant for success in the lexical prediction task in Study 1, in which participants predicted lexical choice of speakers of different ages. Network Density, in contrast, was hypothesized to reduce the variability in the network, as people who regularly interact are likely to converge in their use (Pardo et al., 2012). This could lead the input to be less representative and reflect use by a niche in the population. It might therefore impair success at lexical prediction and lead to less normative lexical choice. As Network Density was not measured in Study 1, we could only assess its influence on own lexical choice.

The results of the studies are in line with the hypothesis that greater variability influences linguistic representations and improves lexical prediction. At the same time, Network Size and Network Density did not exhibit the predicted effects. One possibility is that at the lexical level, Network Size and Network Density do not lead to more variable input. Indeed, Network Density did not correlate with Age Variability, and neither did Network Size.

Needless to say, a network can be variable on many other aspects, from educational level, through geographical distribution to socioeconomic status. It seems likely that such variability would boost performance as well when production varies with these factors. That is, if lexical choice varies with educational level, then having a network that is more heterogeneous in terms of educational level should lead to better prediction. It might also be the case that social network size or network density would correlate with these measures of variability, and thus play a role. Future research should therefore investigate other types of variability. In the meantime, the studies reported here provide preliminary evidence that variability in input could influence production and processing, such that more variable input facilitates lexical access and improves prediction of others’ lexical choices.

One caveat is that these studies did not manipulate people’s social network, but examined associations between properties of existing social networks and performance. It could therefore be the case that the two factors are not causally related or even that it is success at prediction and lexical access ease that determine people’s social networks. While we cannot rule out this possibility, the fact that it is specifically network’s age variability that matters and not other aspects of one’s network reduces the likelihood of this possibility, as it is difficult, for example, to envisage why it is that lexical access leads necessarily to having more age-heterogeneous network. Furthermore, our simulations do not suffer from this confound, and they show that having a more age-variable network leads to input with reduced entropy, which should facilitate lexical access and might similarly improve retrieval during lexical prediction. Nonetheless, further research is required to establish whether the links between age variability in one’s network and ease of lexical access and accuracy of lexical prediction are causal and what their directionality is. For now, we know that those who have more heterogeneous networks, at least in terms of age, show benefits in language use, at least when it comes to lexical access and lexical prediction.

Another caveat is that our social network measure relies on self-report. Self-report measures are often more noisy than precise measurement. For example, it could be that some people are more accurate in their estimates than others. It is possible then that some of the network properties that we did not find to be predictive (e.g., network size) would be found to be predictive using more objective measures. At the same time, such noise cannot account for the patterns that we did find. It similarly seems unlikely that people would intentionally and systematically manipulate the information they provide about the age heterogeneity in their social network, so while we cannot rule this option out completely, it is not immediate clear how it would account for our results.

The results of this study suggest that production and processing might be related, as age variability exhibited influence on both lexical prediction and lexical access. While this does not necessarily mean that the two are related, the results are suggestive and point to the entropy in the distribution as a potential cause. At the same time, the results are at odds with arguments that own use is related to processing of others’ speech, either by covert simulation, and/or by predicting others’ use by first anchoring in one’s own use, and then adjusting. Had this been the underlying mechanism, we would expect age variability in one’s network to also influence lexical choice, yet this was not the case. One caveat is that examination of the Dutch norms that we collected revealed that in Dutch, unlike in English, the dominant names of our stimuli do not vary by age. It is therefore possible that in English, network age variability would influence lexical choice as well. That said, our results do show, though, that network heterogeneity can influence representations and thus facilitate lexical access even without any change in own lexical choice, and thus it could influence prediction even without an influence on lexical choice. Our studies are only the first step in exploring the link between network properties, lexical access and lexical prediction. What we know is that greater age variability reduces entropy in name distribution and the dominance of strong competitors, and these ease lexical access, particularly in cases when lexical access is difficult. The lesser competition from subordinate terms, and the greater ease of selecting a label might strengthen the link between a concept and its label(s), which might facilitate the retrieval and tracking of the names that other people use when labeling objects, improving prediction. Future studies should examine to what degree our findings about off-line lexical prediction also extend to online prediction.

To conclude, this article provides initial evidence for the influence of people’s social network properties on their linguistic performance. It thus opens the door to studying how aspects of our lifestyle might influence the nature of the input we receive, and consequently, our linguistic performance.

## Appendix

**Table 1:** Results of Study 1 (*N* = 86)

	β	*SE*	*z*	*p* value
Intercept	0.211	0.162	1.304	.192
Age range	0.009	0.004	2.667	<.01
Network size	-0.001	0.005	-0.253	.800
Picture agreement	0.024	0.007	3.490	<0.001
Question’s age group	0.858	0.231	3.716	<0.001
Age range × Question’s age group	-0.004	0.005	-0.845	.398
Network size × Question’s age group	0.005	0.007	0.644	.520

**Table 2:** Results of Study 1 (*N* = 86) when the similarity between the age range of a participant’s network and the question’s age group is included

	β	*SE*	*z*	*p* value
Intercept	-1.30	0.54	-2.415	<.02
Age range	0.008	0.004	1.97	<.05
Network size	0.005	0.005	1.12	.26
Picture agreement	0.03	0.008	3.45	<.001
Question’s age similarity to network age	0.25	0.16	1.57	.12
Age range × Question’s age similarity to network age	-0.0004	0.006	-0.07	.94
Network size × Question’s age similarity to network age	-0.02	0.01	-2.06	<.04

**Table 3:** Table of correlations for network measures in Study 2

	Network size	Age range	Network density
Network size	x		
Age range	-0.15	x	
Network density	-0.29	-0.03	x

**Table 4:** Results of Study 2 (*N* = 96)

	β	*SE*	*Z*
Intercept	3.07	0.02	162.13
H index	0.06	0.01	5.41
Response (dominant)	0.03	0.01	-4.86
Age variability	-1.28e-03	1.27e-03	-1.01
Network size	3.13e-04	4.35e-04	0.72
Network density	0.01	0.06	0.19
H Index × Age variability	-1.148e-03	5.553e-04	-2.07
H Index × Network size	-3.90e-05	1.855e-04	-0.21
H Index × Network density	0.01	0.03	0.56

**Table 5:** Results of H Index analysis in Study 3 (*N* = 300)

	β	*SE*	*Z*
Intercept	3.43	0.12	28.42
Age variability	-0.16	0.05	-2.92
Name agreement	-3.30	0.19	-17.43
Age variability × Name agreement	0.23	0.08	2.68

**Table 6:** Results of competition analysis in Study 3 (*N* = 300)

	β	*SE*	*t*
Intercept	-0.95	0.03	-34.71
H Index	0.14	0.004	34.18
Age variability	-0.03	0.01	-2.94
Age variability × H Index	0.02	0.006	3.73

## Footnotes

1 One may wonder whether participants’ own age has an effect, and might even correlate with network age range and underlie the effects we report. To evaluate this possibility, we conducted additional analyses that included age. We had age information for 80 of the participants. Their own age did not correlate with the age range in their network (*r* = -.06, *ns*). More importantly, rerunning the same analysis as reported above while including age leaves the results unchanged. There are still effects of Picture Agreement (β = 0.03, *SE* = 0.01, *z* = 4.27, *p* < .001), Question’s Age Group (β = 1.20, *SE* = 0.51, *z* = 2.34, *p* < .02), and, importantly, Age Range (β = 0.01, *SE* = 0.004, *z* = 2.68, *p* < .01). Participants’ own age has no effect (β = -0.007, *SE* = 0.007, *z* = -1.07, *ns*).

2 As in Study 1, we ran additional analyses to ensure that the effects that we find are not driven by individual differences in participants’ age. In this sample, participants’ own age negative correlated with the age range in their network (*r* = -.21, *p* < .04). To run an analysis with Age, we needed to exclude two participants whose age was more than 2.5 standard deviations from the mean. Additionally, adding Age and its slope into the model led to failure of convergence, so we had to omit the slope that explained the least variance, which was the slope for Network Size. The results of this model shows an effect of Age (β = 0.004, *SE* = 0.002, *t* = 2.1), such that older participants are slower to respond. Importantly, the other effects remain unchanged. There is still an effect of Response (β = -0.03, *SE* = 0.01, *t* = -4.8), an effect of H Index (β = 0.04, *SE* = 0.01, *t* = 5.2), and the crucial interaction of H index with Age Variability (β = -1.1e-3, *SE* = 5.6e-4, *t*= -2).

3 We replaced all failures of recognition (e.g., naming *salt* as *pepper*) with the response *null* to allow collapsing over all misidentifications.
